# Weak linkage between the heaviest rainfall and tallest storms

**DOI:** 10.1038/ncomms7213

**Published:** 2015-02-24

**Authors:** Atsushi Hamada, Yukari N. Takayabu, Chuntao Liu, Edward J. Zipser

**Affiliations:** 1 Atmosphere and Ocean Research Institute, The University of Tokyo, Kashiwa 277-8568, Japan; 2 Department of Physical and Environmental Sciences, Texas A&M University-Corpus Christi, Corpus Christi, Texas 78412-5850, USA; 3 Department of Atmospheric Sciences, University of Utah, Salt Lake City, Utah 84112-0110, USA

## Abstract

Conventionally, the heaviest rainfall has been linked to the tallest, most intense convective storms. However, the global picture of the linkage between extreme rainfall and convection remains unclear. Here we analyse an 11-year record of spaceborne precipitation radar observations and establish that a relatively small fraction of extreme convective events produces extreme rainfall rates in any region of the tropics and subtropics. Robust differences between extreme rainfall and convective events are found in the rainfall characteristics and environmental conditions, irrespective of region; most extreme rainfall events are characterized by less intense convection with intense radar echoes not extending to extremely high altitudes. Rainfall characteristics and environmental conditions both indicate the importance of warm-rain processes in producing extreme rainfall rates. Our results demonstrate that, even in regions where severe convective storms are representative extreme weather events, the heaviest rainfall events are mostly associated with less intense convection.

## Abstract (web summary)

Conventionally, the heaviest rainfall is associated with the most intense storms, yet this relationship remains untested. Here, Hamada *et al*. analyse 11 years of radar observations from the topics and subtropics, and conclude that the heaviest rainfall is most commonly associated with less intense convection.

## Graphical abstract

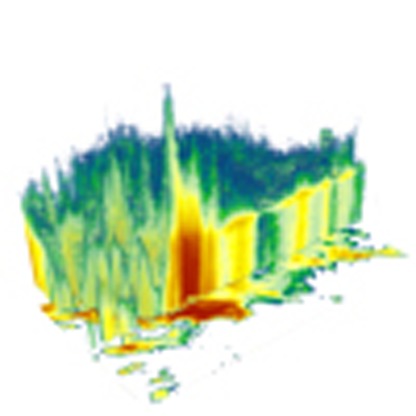

The intensity of instantaneous rainfall rate has conventionally been linked to the intensity of convective storms. There is an extensive literature on the characteristics of severe storms on both global and regional scales, in which these storms are usually characterized as having extremely strong convection. On the basis of spaceborne radar and radiometer measurements, the global distribution of extreme convective events has been relatively well described^1^,^2^,^3^,^4^. There are also many detailed regional studies on the rainfall characteristics of extreme convective events^5^,^6^,^7^.

On the other hand, there are observational facts that certain types of extreme rainfall events, such as flash flooding storms, do not necessarily accompany extremely strong convection and intense lightning activity, even in regions where severe convective storms are representative extreme weather events^8^,^9^,^10^,^11^,^12^. In some regions, heavy surface rainfall is typically associated with relatively low-echo-top heights^13^,^14^,^15^. Moreover, heavy orographic rainfall can inherently exhibit lower echo-top heights^16^. There are also observations which show that convective intensity is not necessarily related to the near-surface rainfall intensity in some specific regions^17^,^18^,^19^.

Extreme rainfall over short durations could have devastating socioeconomic effects, as one of the key factors of flash flooding. Therefore, such a decorrelation between the top heights of strong echoes and heavy/extreme surface rainfall rates highlights the importance of a detailed study that focuses on the inherent characteristics of extreme rainfall events and contrasts those with extreme convective events.

Despite many detailed regional studies, a global picture of the linkage between extreme convection and extreme surface rainfall rate remains unclear. Here we use a unique long-term record from a spaceborne precipitation radar (PR) to investigate the characteristics of rainfall events that produce the greatest instantaneous rainfall rates in the tropics and subtropics. We further demonstrate statistically a weak linkage between the extreme rainfall events and extreme convective events. Furthermore, the differences in rainfall characteristics between extreme rainfall events and extreme convective events and their regionality are investigated.

## Results

### Robust differences between two extremes

**Figure 1:**
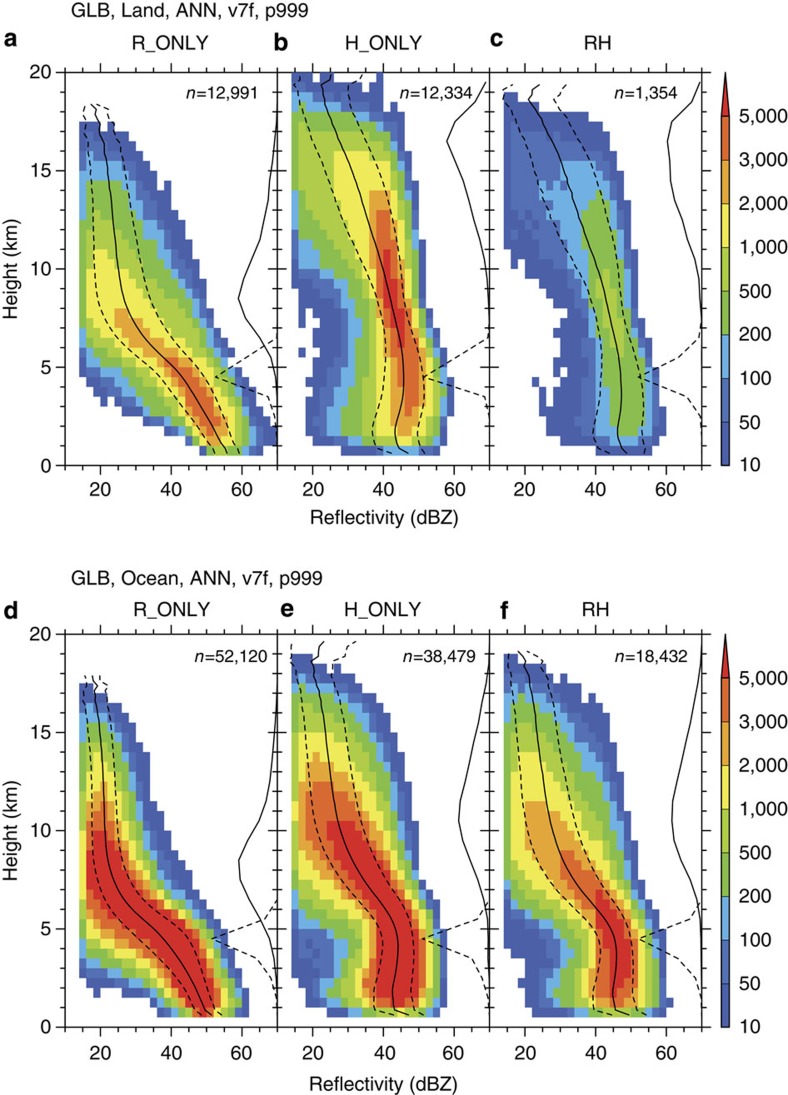
Echo profiles for three types of extreme events. Composite structures of radar reflectivity at extreme pixels within the TRMM observation domain (37.5°S–37.5°N). Colours show joint histograms of effective radar reflectivity and height, superimposed by solid and dashed lines that indicate the mean and s.d. for each height bin, respectively. Solid and dashed lines along the right-hand axis of each panel show the histograms of echo-top heights and 0 °C levels, respectively. The number of samples for the corresponding extreme type is indicated. (**a**–**c**) R-only, H-only and RH-extreme events over land, respectively. (**d**–**f**) R-only, H-only and RH-extreme events over the ocean, respectively.

Figure 1 shows composite structures of effective radar reflectivity (Ze) at extreme pixels where the maximum near-surface rainfall rate or maximum 40-dBZ echo-top height is observed for each type of extreme event for land and the ocean. There are clear and robust differences in the vertical structures between R-only and H-only extreme events. Note that the rain type, that is, convective or stratiform, is not considered, because more than 95% of the extreme pixels were determined as convective for all extreme types. Clear differences are observed in echo profiles between R-only and H-only extreme events, both over land and over the oceans, since the differences are more significant over land than over the ocean. The most striking characteristic is the echo structure of R-only extreme events both over land and over the oceans (Fig. 1a,d); the echo-top heights, which are defined as the maximum height of the precipitation rate of 0.5 mm per hour in each rain event, are relatively low (around 8–9 km). The reflectivity profile exhibits a large and almost linear downward increase below 8 km. The vertical gradient of reflectivity tends to be larger around and just above the melting level at 4–5 km. Such a large linear downward increase is obviously related to significant downward increase of rainfall rates (Supplementary Fig. 2). These characteristics of R-only extreme event profiles show very little difference between land and ocean, except that on average, reflectivities over land are ~5 dB greater than over the ocean at all heights.

Echo structures of H-only extreme events are significantly different from those of R-only extreme events, both over land and the ocean (Fig. 1b,e). Distributions of the maximum echo-top height of H-only extreme events have maxima around 16–17 and 10–11 km over land and the ocean, respectively. While it is not surprising that the maximum echo-top heights of H-only extreme events are higher than those of R-only extreme events by definition, their large discrepancy over land is worth noting. The reflectivity profiles exhibit structures that are aligned more vertically and that show a slight downward decrease below the melting level, compared with R-only extreme events.

RH-extreme events exhibit echo structures similar to H-only extreme events. An interesting feature of RH-extreme events is that even though they are defined both as extreme rainfall and as extreme convective events, their near-surface reflectivities or rainfall rates are somewhat lower than those of R-only extreme events.

**Figure 2:**
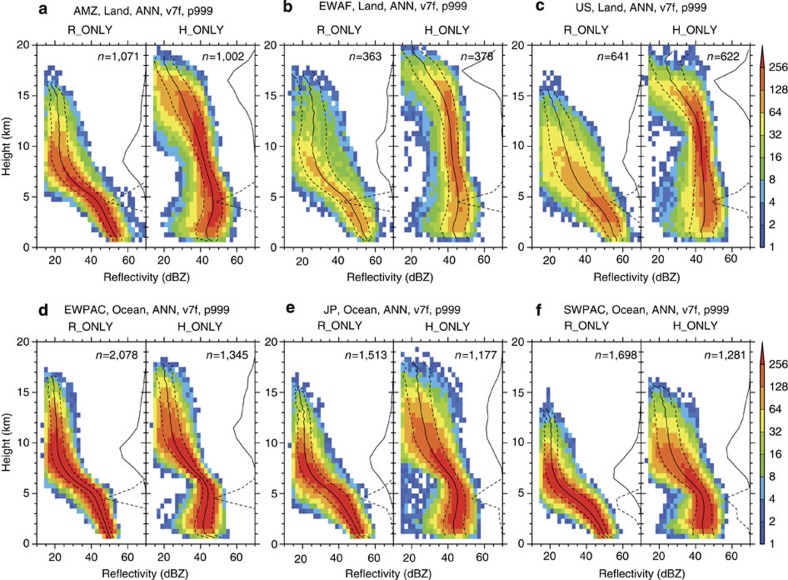
Regional variation of extreme event characteristics. The same as in Fig. 1, but for several specific geographical regions. Only the panels for R-only and H-only extreme events are shown for each region. (**a**) Amazon (70°W–40°W, 15°S–0°, over land). (**b**) Equatorial western Africa (15°W–15°E, 0°–15°N, over land). (**c**) Southern North America (110°W–80°W, 22°N–37°N, over land). (**d**) Equatorial western Pacific (140°E–170°E, 0°–15°N, over ocean). (**e**) Northwestern Pacific around Japan (120°E–150°E, 22°N–37°N, over ocean). (**f**) Southwestern Pacific (180°–150°W, 37°S–22°S, over ocean).

Interestingly, the differences in echo structures between R-only and H-only extreme events are robust with respect to region. Fig. 2a–f shows similar features as Fig. 1, but for several specific geographical regions. The first three panels depict the results obtained over land: the Amazon, equatorial western Africa and around the United States (Fig. 2a–c). The final three panels depict the results obtained over oceanic regions: the equatorial western Pacific, northwestern Pacific around Japan and southwestern Pacific (Fig. 2d–f). The characteristics found in the global composite (Fig. 1) can be observed in all of the specific regions, demonstrating that the differences in the characteristics between the R-only and H-only profiles are not unique to any particular geographical area. The echo structures of R-only extreme events show very little regional variation over either land or ocean, whereas those of H-only extreme events do present some regional variations in the upper troposphere. This robustness can be confirmed statistically by illustrating the global mean reflectivity profiles of R-only and H-only extreme events (Supplementary Fig. 3). It is demonstrated that extreme rainfall rate is mostly associated with R-only-type rainfall systems, even in regions where severe convective storms are dominant, such as the central United States^5^.

### Weak linkage between heaviest rainfall and tallest storms

**Figure 3:**
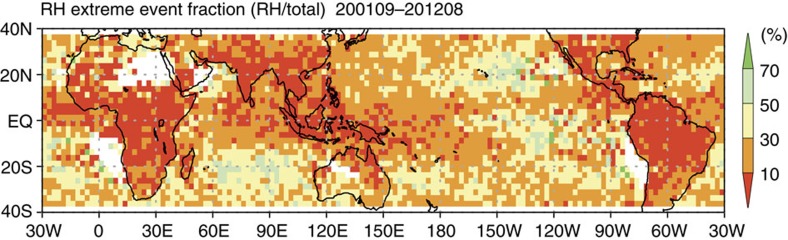
Weak linkage between extreme rainfall and convective events. Fraction of the number of RH-extreme events to that of total extreme events in each region.

Another notable feature shown in Fig. 1 is that only a very small fraction of regional extreme events is categorized as RH-extreme events. Figure 3 shows the ratio of the number of RH-extreme events to the total number of regional extreme events in each region, illustrating the extent of the linkage between extreme rainfall and extreme convective events; a value of 100 means all the extreme rainfall and extreme convective events are identical. It is clear that the ratio is very low in most of the region of Tropical Rainfall Measurement Mission (TRMM) observation, indicating very little correlation between extreme rainfall events and extreme convective events. Over land in particular, the ratio is less than 30% almost everywhere. This ratio is even lower in the deep tropics where less than 10% of extreme rainfall events are related to extreme convective events. Over the oceans, the ratio is slightly higher than over land, but it remains less than 50% in most regions. The ratio tends to be lower in rainy regions such as the tropical warm pool and Intertropical Convergence Zone, while it is slightly higher in suppressed regions such as the eastern subtropical Pacific. This weak linkage between extreme convective and extreme rainfall events results in large differences in rainfall rates and the maximum height of strong echoes produced by each type of extreme event (Supplementary Fig. 4). Extreme convective events produce surface rainfall rates only less than half of extreme rainfall events, in most land regions and also in many parts of the oceans. When comparing the size distributions of extreme rainfall and convective events, the median value of event size for extreme rainfall events is larger than that for extreme convective events with statistical significance at 95% confidence level using Mann–Whitney *U*-test.

The weak linkage can partly be attributed to clear seasonal differences in the occurrences of R-only and H-only extreme events (Supplementary Fig. 5). In most regions, monthly occurrence frequencies of R-only extreme events are almost in phase with monthly variation of total rainfall in the region. However, those of H-only extreme events tend to have shorter-period peaks different from the rainiest season.

### Differences in the related environmental conditions

**Figure 4:**
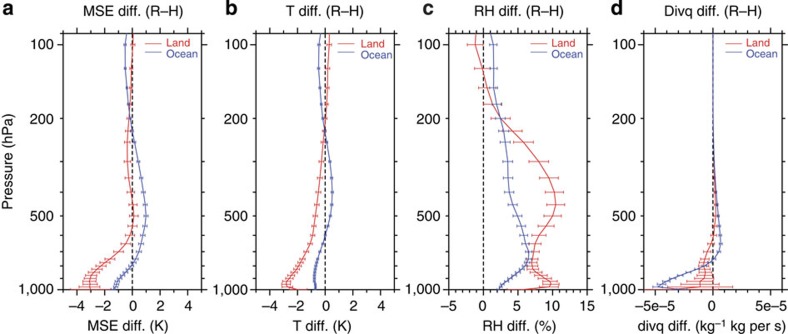
Environmental profile differences. Vertical profiles of the differences between R-only and H-only extreme events. Red and blue colours show the difference over land and the oceans, respectively. Solid lines and error bars show the mean and 99% confidence intervals based on the *t*-statistic at each height, respectively. (**a**) Moist static energy (in units of K). (**b**) Temperature. (**c**) Relative humidity. (**d**) Moisture flux divergence.

Environment also shows robust differences between R-only and H-only extreme events. Figure 4 shows the mean difference in atmospheric stratifications of environments for R-only and H-only extreme events and their 99% confidence intervals at each pressure level. The averages and confidence intervals were obtained using the mean values at 2.5 × 2.5°-grid cells, calculated using the environmental profiles taken from ERA-interim reanalysis data. The differences of moist static energy (divided by the specific heat of air at constant pressure; in units of K) and temperature between R-only and H-only extreme events are mostly negative, but exhibit positive vertical gradients in the lower troposphere both over land and over the oceans, indicating that R-only extreme events are found in less convectively unstable environments than H-only extreme events (Fig. 4a,b). Relative humidity differences have positive values from the surface to around 200 hPa, indicating wetter environments for R-only extreme events than for H-only events (Fig. 4c). The differences of moisture flux divergence (Fig. 4d) exhibit a signature that is more convergent in the lower troposphere for R-only extreme events, especially over the oceans. These differences might also be attributed to typical environmental differences between active and break periods of the monsoon^20^, which are considered the cause of the variations of convective properties within the monsoon^21^,^22^.

## Discussion

The differences between the echo structures of R-only and H-only extreme events could be interpreted in terms of the differences of their related environmental characteristics. A unique characteristic of the echo structure for R-only extreme events, that is, lower echo-top height with large downward increase of reflectivity around the melting level, resembles the so-called ‘low-echo centroid’ structure, which is often observed in excessive rainfall events related to flash floods^10^,^11^,^12^. The low-echo centroid structure is considered associated with convection where the collision–coalescence process is dominant in precipitation production. A descending core in a hailstorm could also appear as similar bottom-heavy echo structure, but the clear difference in seasonal occurrences of extreme rainfall and convective events supports different rainfall production processes as a main cause for the different echo structures. Events of excessive rainfall from warm-rain processes, which are typically more efficient than cold-rain processes^23^, require a deep warm-cloud layer (that is, the layer from the lifting condensation level to the freezing level), moderate updrafts, limited wind shear, and high relative humidity through a deep layer^24^. The differences in the environments between R-only and H-only extreme events might indicate that R-only extreme events are mostly associated with the efficient production of precipitation by collision–coalescence, which is dominant in warm-rain processes.

Our key finding is that such observed differences in rainfall characteristics and related environments are robust throughout the tropics and subtropics, and that even in regions where severe convective storms are representative extreme weather events, the most extreme rainfall events are more closely related with less intense convection. These findings will help improve the understanding of the physical processes of extreme rainfall.

## Methods

### Data

We used a rainfall-event database based on TRMM PR measurements that has been developed by the authors^15^,^25^. The database contains rainfall and echo characteristics of rainfall events, which are defined as a set of contiguous pixels with a near-surface rainfall rate >0.5 mm per hour based on the TRMM PR 2A25 product^26^,^27^. Using a rainfall event as the sampling unit, rather than individual rainy pixels, other characteristic values such as the maxima of surface rainfall rate and echo-top height can be obtained for each event. In this study, we used the rainfall-event database based on the 2A25 version 7 product. A filter for the removal of false-extreme rainfall related to contamination by ground or mainlobe clutter was applied before constructing the rainfall-event database^25^. Although the 2A25 product and the rainfall-event database are available for a 16-year period from December 1997, we used the section of the database from September 2001 to August 2012 in this study to avoid the influence of altitude increase of the TRMM satellite in August 2001 on PR sensitivity^28^.

Environmental atmospheric profiles related to the extreme events were calculated using the European Centre for Medium-Range Weather Forecasts Reanalysis Interim (ERA-interim) data set^29^. An environmental profile related to an extreme event was defined as a vertical profile of the ERA-interim data set that was nearest in time to the observed time of the extreme event and spatially interpolated to the location of the extreme pixel.

We must register an important caution that could affect the accuracy of the PR reflectivity profiles, particularly for the H-only events, with very high radar reflectivity aloft. It is well known that there are uncertainties in the evaluation of the total path attenuation of the TRMM Ku-band radar beam, and how this total path attenuation is distributed in the vertical. This issue has been extensively discussed^26^,^27^,^30^ by providers and users of the PR data, and we continue to evaluate this issue; however, we believe that it does not alter the sense of our principal results.

### Definition of extreme events

We analysed measurements made over an 11-year period by the PR onboard the TRMM satellite. Here a ‘rainfall event’ is defined as a set of contiguous rainy pixels at near-surface level. Such an event-oriented method facilitates the derivation of other characteristic values such as the maxima of surface rainfall rate and echo-top height. We first accumulated rain events for each 2.5 × 2.5° grid cell within the region of TRMM observation. Subsequently, regional extreme rainfall events in each grid cell were defined as those with maximum near-surface rainfall rates within the uppermost 0.1%. Extreme convective events were defined in a similar manner using the maximum 40-dBZ echo-top heights instead of near-surface rainfall rate. Having defined the regional extreme rainfall and convective events separately, they were categorized into one of three types: R-only extreme, determined as an extreme rainfall event, but not an extreme convective event; H-only extreme, determined as an extreme convective event, but not an extreme rainfall event; and RH extreme, determined as both an extreme rainfall and extreme convective event. By definition, the numbers of R+RH and H+RH extreme events should be identical, although in reality there is a slight difference between the two numbers, mainly because of quantization of echo-top heights in 250-m vertical bins. So, we could have used either R+RH or H+RH for the total number. In this study, we selected R+RH-extreme events for the number of regional extreme events (Supplementary Fig. 1).

## Author contributions

A.H. and Y.N.T. conceived and designed the study; A.H. performed most of the analyses and wrote the manuscript discussing with Y.N.T.; and all the authors took part in the discussions of the results and contributed to the manuscript.

## Additional information

**How to cite this article:** Hamada, A. *et al*. Weak linkage between the heaviest rainfall and tallest storms. *Nat. Commun.* 6:6213 doi: 10.1038/ncomms7213 (2015).

## Supplementary Material

Supplementary Information Supplementary Figures 1-5
