# Supplementary material for: Weak linkage between the heaviest rainfall and tallest storms
Source: Nat Commun. 2015 Feb 24;6:6213. doi: 10.1038/ncomms7213 (PMC4346623; doi:10.1038/ncomms7213)
Supplement: Supplementary Information — Supplementary Figures 1-5 [file ncomms7213-s1.pdf]

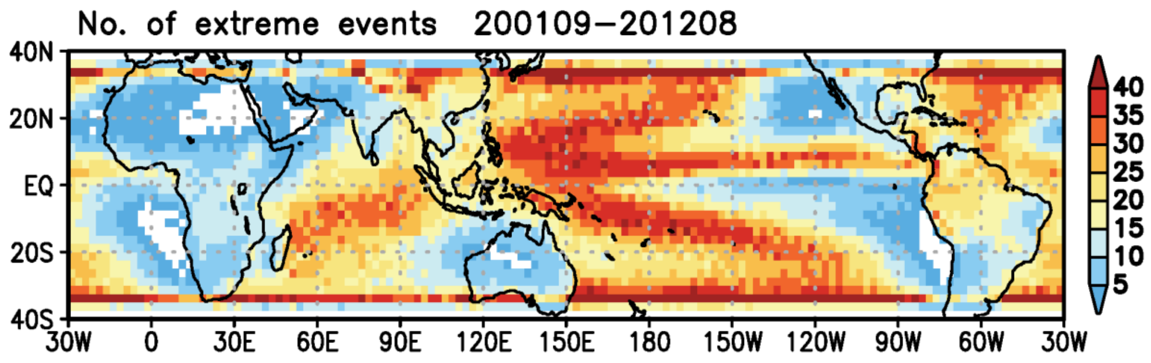

Supplementary Figure 1. Number of total extreme events in each region which isdefined as the sum of the numbers of the R-only and RH extreme events, corresponding tobasically 0.01% of the total rainfall events within each region.The number of extremes, as well as total, peaks near 35°N/S, because the orbit passes more at these latitudes due to the orbit inclination.

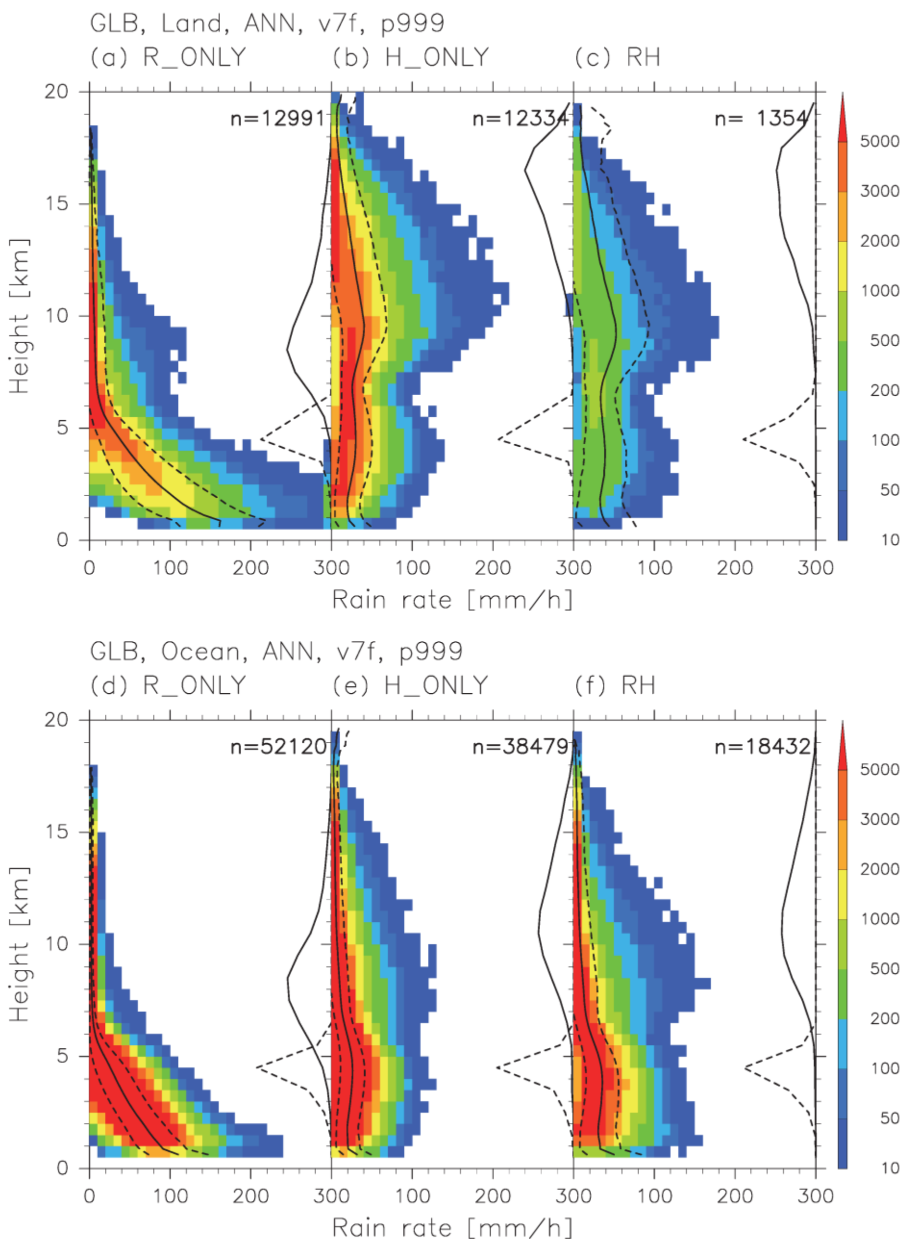

Supplementary Figure 2. Composite structures of radar reflectivity at extreme pixels within the TRMM observation domain (37.5°S–37.5°N). Colors show joint histograms of precipitation rate (mm h^{-1}) and height, superimposed by solid and dashed lines that indicate the mean and standard deviation for each height bin, respectively. Solid and dashed lines along the right-hand axis of each panel show the histograms of echo-top heights and 0°C levels, respectively. The number of samples for the corresponding extreme type is indicated. (a–c)R-only, H-only, and RH extreme events over land, respectively.(d–f)R-only, H-only, and RH extreme events over the ocean, respectively.

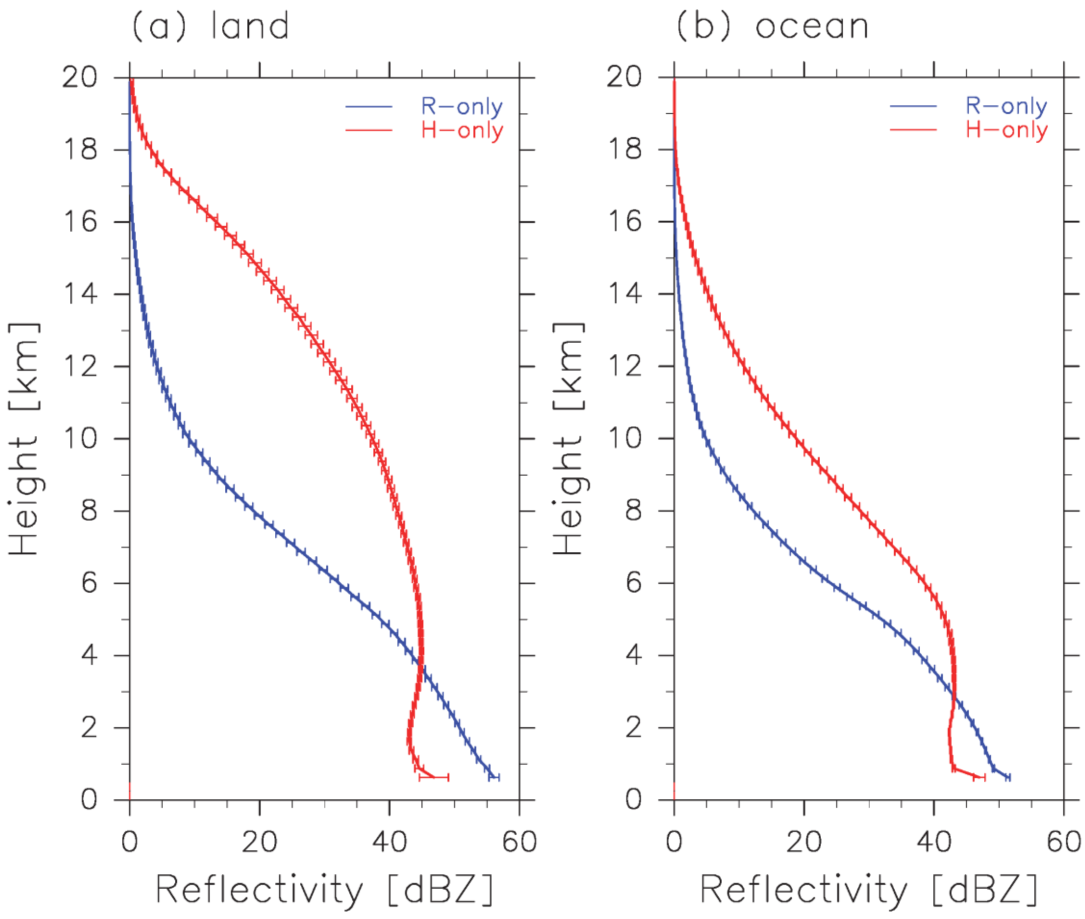

Supplementary Figure 3. Vertical profiles of mean radar reflectivity for R-only (blue) and H-only (red) extreme events over (a) land and (b) the ocean.To mitigate the spatially inhomogeneous sampling of the precipitation radar, the averages and confidence intervals are calculated using the mean reflectivities at 2.5° × 2.5°-gridcells, which have been calculated from the reflectivity profiles of R-only or H-only extreme events in the corresponding gridcell. Error bars show the 99% confidence intervals based on the t-statistics.

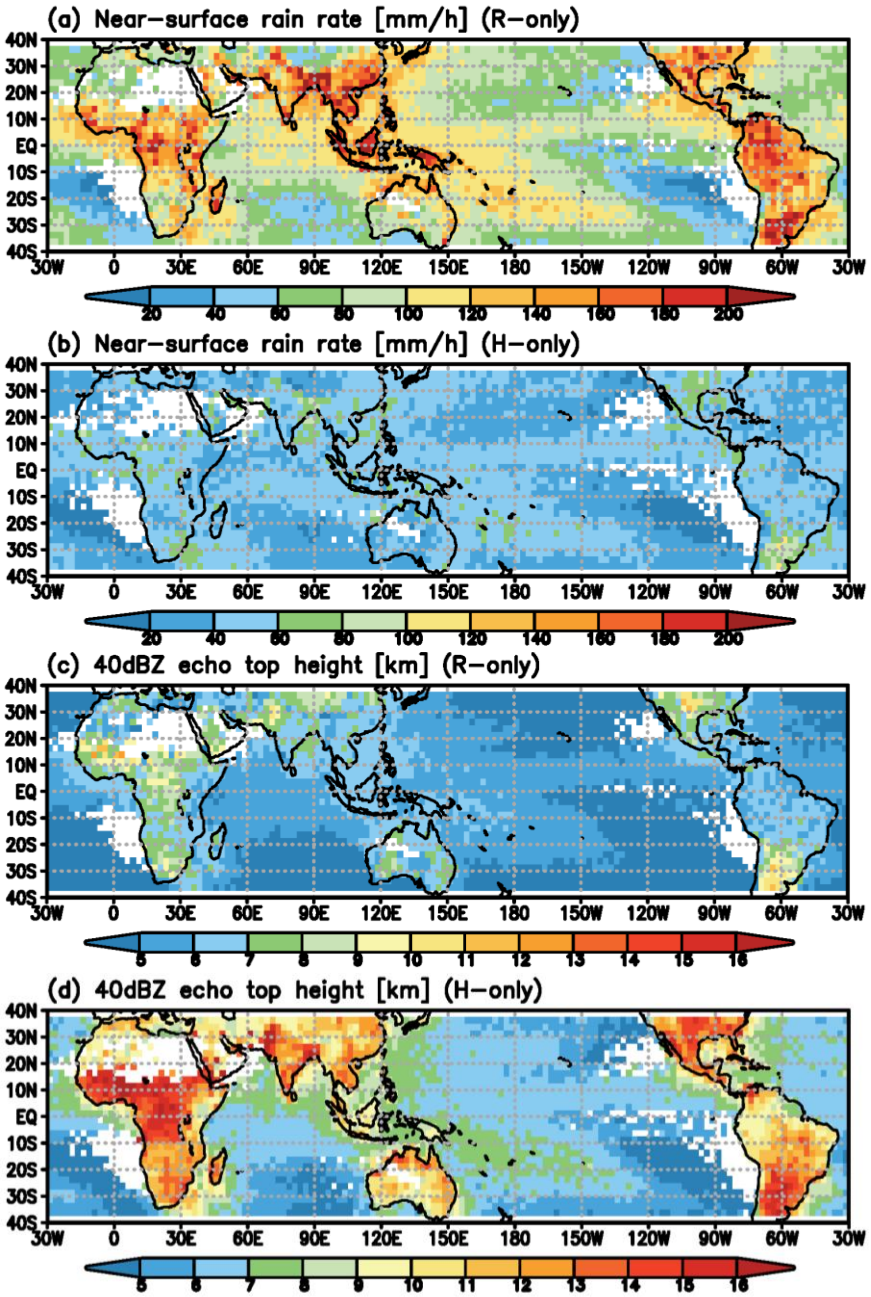

Supplementary Figure 4. Distribution of (a,b) maximum near-surface rainfall rate and (c,d) maximum 40-dBZ echo top heights for (a,c) R-only extreme events and (b,d) H-only extreme events. Values are shown as averages for all corresponding types of extreme events at individual gridcells. Gridcells in which the total number of extreme events is less than three are not colored.

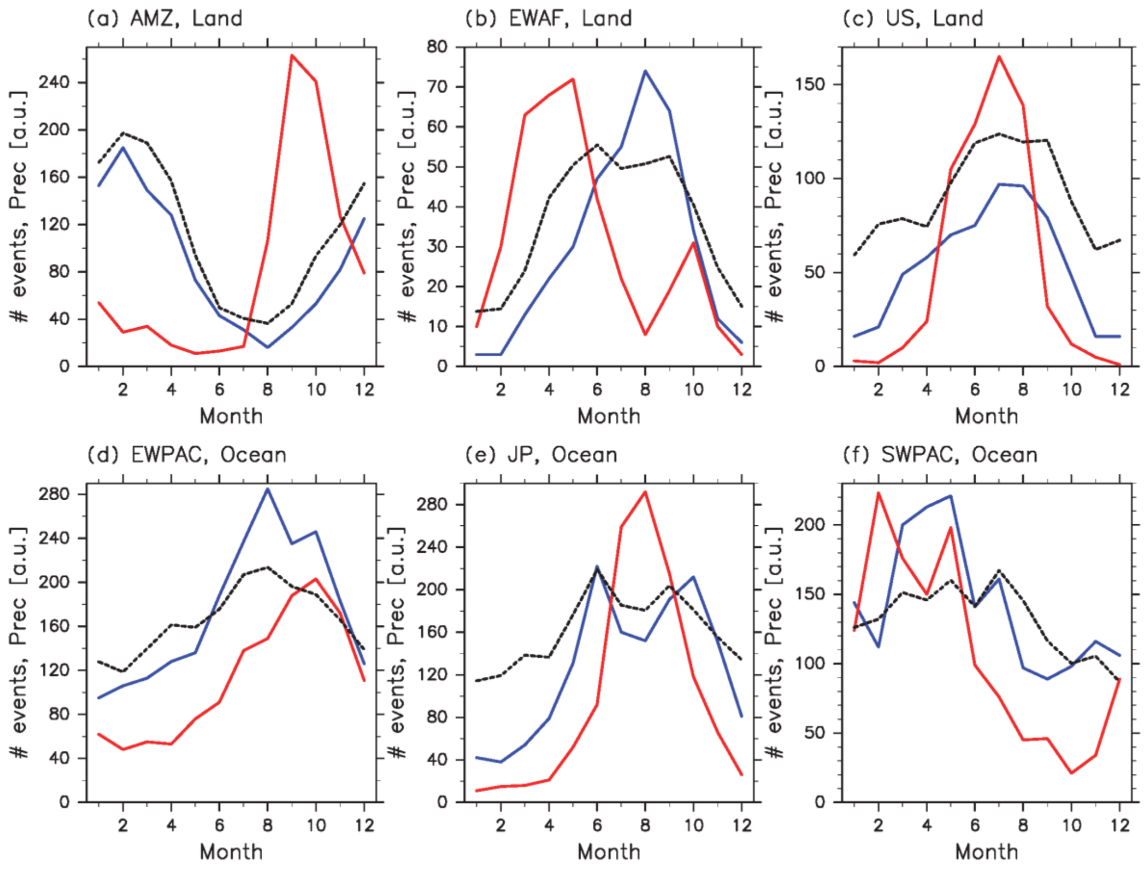

Supplementary Figure 5. Monthly occurrence frequencies of R-only (blue) and H-only (red) extreme events, for the specific regions shown in Fig. 2. Black dashed lines show monthly precipitation amount which are calculated using TRMM 3A25 data set from 2001 to 2012and have been normalized adequately for visualization.
